# The role of exosomes in liver cancer: comprehensive insights from biological function to therapeutic applications

**DOI:** 10.3389/fimmu.2024.1473030

**Published:** 2024-10-21

**Authors:** Yinghui Zhang, Congcong Zhang, Nan Wu, Yuan Feng, Jiayi Wang, Liangliang Ma, Yulong Chen

**Affiliations:** ^1^ College of Rehabilitation Medicine, Henan University of Traditional Chinese Medicine, Zhengzhou, Henan, China; ^2^ Rehabilitation Center, The First Affiliated Hospital of Henan University of Chinese Medicine, Zhengzhou, Henan, China

**Keywords:** exosomes, liver cancer, biological functions, diagnostic biomarker, immunosuppression

## Abstract

In recent years, cancer, especially primary liver cancer (including hepatocellular carcinoma and intrahepatic cholangiocarcinoma), has posed a serious threat to human health. In the field of liver cancer, exosomes play an important role in liver cancer initiation, metastasis and interaction with the tumor microenvironment. Exosomes are a class of nanoscale extracellular vesicles (EVs)secreted by most cells and rich in bioactive molecules, including RNA, proteins and lipids, that mediate intercellular communication during physiological and pathological processes. This review reviews the multiple roles of exosomes in liver cancer, including the initiation, progression, and metastasis of liver cancer, as well as their effects on angiogenesis, epithelial-mesenchymal transformation (EMT), immune evasion, and drug resistance. Exosomes have great potential as biomarkers for liver cancer diagnosis and prognosis because they carry specific molecular markers that facilitate early detection and evaluation of treatment outcomes. In addition, exosomes, as a new type of drug delivery vector, have unique advantages in the targeted therapy of liver cancer and provide a new strategy for the treatment of liver cancer. The challenges and prospects of exosome-based immunotherapy in the treatment of liver cancer were also discussed. However, challenges such as the standardization of isolation techniques and the scalability of therapeutic applications remain significant hurdles.

## 1 Introduction

In recent years, the incidence of cancer has been increasing, posing a major threat to human health and life safety. According to the 2024 Global Cancer Statistics, the survival rate for liver cancer is only 22%. Liver cancer includes hepatocellular carcinoma (HCC), intrahepatic cholangiocarcinoma, cholangiocarcinoma, and fibrolamellar carcinoma. Most of them are HCC. Primary liver cancer has become the fifth leading cause of cancer diagnosis in the world and the third leading cause of cancer death threatening human health (1). Currently, clinical treatments for liver cancer primarily include surgery, radiotherapy, and chemotherapy. However, liver cancer cells may evade the attack of human immune system and make themselves survive and proliferation in the host (2). In the context of hepatocellular carcinoma treatment, while surgery is commonly employed for tumor eradication, the presence of underlying conditions such as liver cirrhosis in most patients elevates surgical risks and the likelihood of recurrence (3, 4).

Recent studies have pointed to EVs, especially exosomes, as potential biomarkers for the diagnosis of liver cancer (5, 6). As the “carrier” of intercellular communication, exosomes are stable and rich in composition, which can effectively convey tumor information, including cell origin and gene mutation (7, 8). In the context of liver cancer, exosomes play pivotal roles by transmitting various bioactive molecules that regulate cancer cell proliferation, invasion, and metastasis processes (9, 10). The detection of exosomes in the blood of patients can achieve early diagnosis, condition monitoring, and prognosis assessment of liver cancer (11). Exosomes also hold promise in liver cancer therapy, serving as carriers for drug delivery to achieve precise treatment and potentially for immunotherapy applications by modulating immune cell activity within the tumor microenvironment to enhance anti-tumor immune responses (9, 12, 13). Liver cancer stem cell-derived exosomes in the microenvironment promote the growth, progression and metastasis of HCC by reducing cell apoptosis, increasing angiogenesis, enhancing metastatic invasiveness and inducing epithelial-mesenchymal transition (14, 15). Research on exosomes offer novel directions and hope for the diagnosis and treatment of liver cancer.

This review reviews the multiple roles of exosomes in liver cancer, including the initiation, progression, and metastasis of liver cancer, as well as their effects on angiogenesis, epithelial mesenchymal transformation (EMT), immune evasion, and drug resistance. This review aims to fill the knowledge gap in the current research on the biological functions and clinical applications of exosomes in liver cancer, delving into the regulatory mechanisms, signal transduction pathways, and potential clinical applications of exosomes in the process of liver cancer development and progression, providing new insights and directions for future research and clinical practice.

## 2 Formation, release and uptake of exosomes

### 2.1.Exosome formation

Exosomal formation is a complex and delicate process involving many cellular mechanisms (16, 17). The formation of exosomes begins with endocytosis within the cell, leading to the formation of early endosomes through invagination. Then, early endosomes fuse with lysosomes to form multivesicular bodies (MVBs) (18). MVBs is the central site of exosome biogenesis. Typically, MVBs originate from endocytosis, and different mechanisms mediate the outgrowth of the plasma membrane and the formation of early endosomes. MVBs formation involves mechanisms of plasma membrane growth and early endosome formation, and cells decide which proteins and other molecules should be included in exosomes through mechanisms such as ESCRT complexes (19). After MVBs fuses with cell membrane, exosomes are released, and this process needs precise regulation. The released exosomes can spread with body fluids to other cells and tissues and participate in long-term regulation of immune response, tissue repair and disease progression. Recent studies have shown that different cellular stress factors such as hypoxia and oxidative stress can significantly affect the release process of exosomes. After stress stimulation, cells will regulate the generation and release of exosomes, which in turn affects the type and content of bioactive molecules carried by exosomes (20). In addition, studies on post-translational modifications of exosomes have also shown that glycosylation, phosphorylation and other modifications can affect the interaction and uptake efficiency of exosomes with target cells (21).

**Figure 1:**
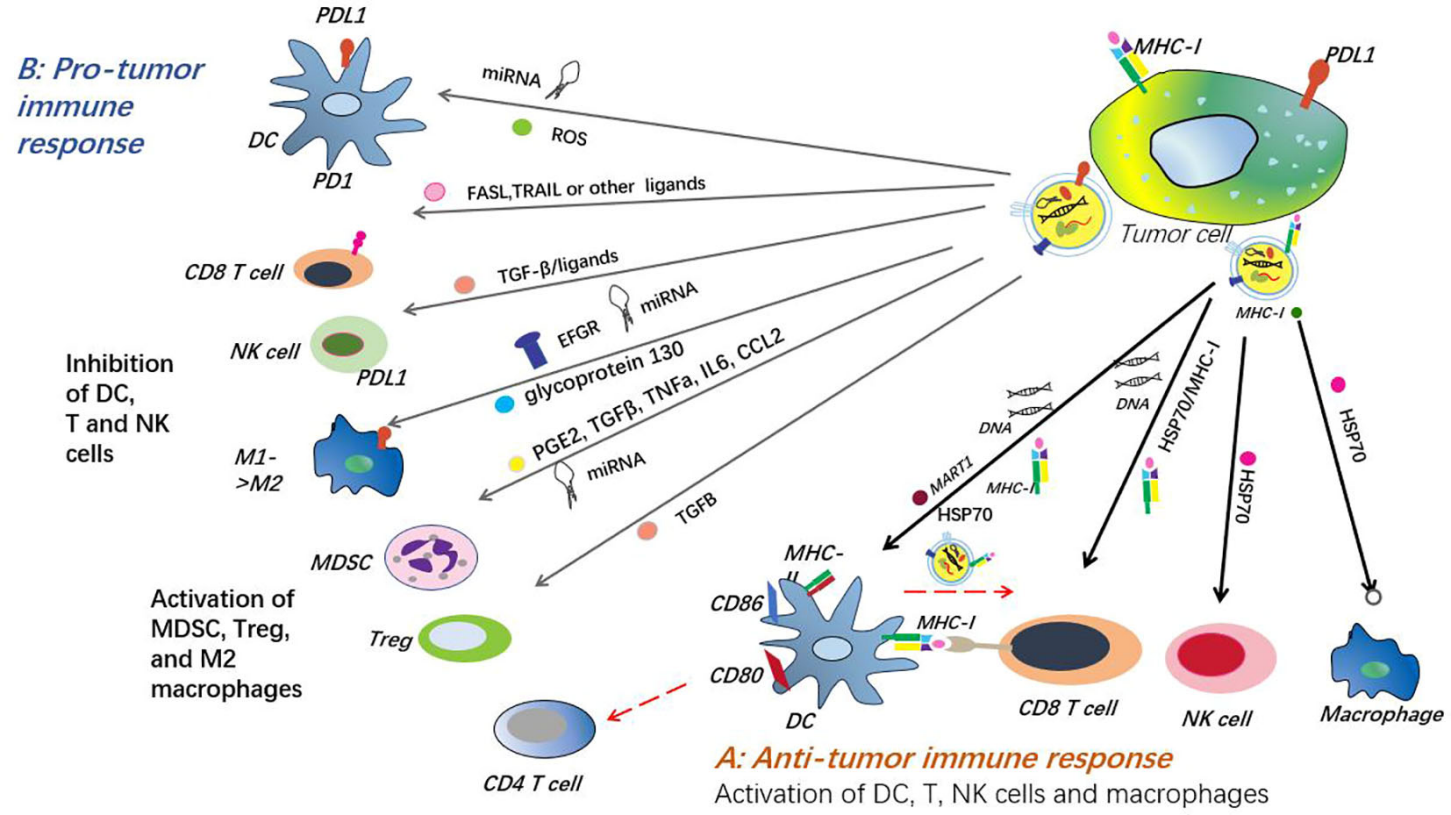
Exosome formation, release and absorption. The formation of exosomes begins with membrane invagination to form endosomes, which then develop into multivesicular bodies (MVBs) containing multiple intracavitary vesicles (ILVS). This process is regulated by a variety of proteins and enzymes, ensuring proper assembly of exosomes. Polyvesicles (MVB) have two fates: fusion with lysosomes leading to ILV degradation, or fusion with the plasma membrane of the cell to release ILV outside the cell to form exosomes. The release of exosomes is strictly regulated and involves a variety of proteins and molecular mechanisms.

In summary, the formation of exosomes begins with endocytosis, forming early endosomes, and then fusing with lysosomes to form MVBs, which is the core of their biogenesis. When MVBs is formed, cells use mechanisms such as ESCRT complexes to sort goods into exosomes. Ultimately, MVBs fuses with cell membranes to precisely regulate exosome release and participate in a variety of biological processes (**Figure 1**).

### 2.2 Exosome release and absorption

Multivesicular bodies release exosomes through budding, a process that requires the involvement of vesicle transport and fusion mechanisms. The internalizing selectivity complex (ESCRT) and its related proteins, such as basket proteins, TSG101 and Alix, are necessary for this process.The ESCRT system promotes the invagination of endosomal membrane and the formation of ILVs through a series of complex molecular interactions and membrane reorganization processes (22). However, to achieve cargo sorting and ILV formation, the ESCRT complex alone is not sufficient; assistance from sphingolipid ceramide is required. In addition,Some studies have shown that shown that tetranester proteins play an important role in the biogenesis and secretion of exosomes independent of the ESCRT mechanism (23). Exosome release and absorption involves the mediation of a variety of cell surface receptors, such as receptor tyrosine kinases and G-protein-coupled receptors. Exosomes are released into the extracellular space through mechanisms such as binding to surface receptors of target cells, membrane fusion with target cells, and endocytosis by target cells.Subsequently, exosomes are absorbed by other cells into their endosomes, or lysosomes, where the contents are released and put to use (24).

In summary, exosomes are absorbed by target cells through a series of complex and precise mechanisms after being released. This includes specific binding to cell surface receptors, membrane fusion, and endocytosis.

## 3 Isolation and purification of exosomes

The study of exosomes is faced with the problem of low yield and insufficient purity. The separation techniques should be carefully selected according to the study objectives and sample characteristics (25).

The isolation and purification of exosomes refers to the various methods and technologies used to separate and purify exosomes from cell culture supernatants and biological fluids. The isolation and purification of exosomes involve techniques like ultracentrifugation, commercial kits (e.g., ExoQuick™), immunomagnetic bead separation, ultrafiltration, sucrose gradient centrifugation, polymer enrichment, size exclusion, field flow fractionation, microfluidic chips (26, 27). But all of them have certain limitations. Ultracentrifugation is a commonly used separation and purification method, and the use may lead to co-purification of non-exosome particles, which may affect the accuracy and interpretation of downstream applications. Ultracentrifugation works by separating particles based on their size and density differences under centrifugal force. However, due to the wide range of exosome sizes, about 30 to 150 nm, and the overlap with other EVs and protein particles, non-exosome particles easily settle down with the exosomes during ultracentriollation (28). The ExoQuick™ kit may isolate at a relatively low purity and may contain non-exosomal components. Because the separation method is mainly based on polymer precipitation method, the obtained exosome samples may contain more non-exosome components such as proteins and lipid particles, with low purity. This lack of purity may interfere with high-precision molecular analyses such as proteomics or RNA sequencing (29). Immunomagnetic bead separation method separates exosomes by specifically binding the antigens on the surface of exosomes with specific antibodies, achieving rapid separation of exosomes, but the cost is relatively higher and requires specific antibodies and magnetic beads (30). Ultrafiltration, a classical method for exosome isolation, is suitable for purifying exosomes from large volumes of culture medium. It relies on size differences to separate exosomes, offering a simple process without complex steps or expensive reagents. Ultrafiltration efficiently preserves the integrity and biological activity of exosomes, enabling large-scale preparation for clinical research and applications. However, smaller exosomes may be lost during filtration, depending on the choice and performance of the filter membrane. Advanced methods such as microfluidics use micrometer scale fluid manipulation to efficiently separate exosomes by their physical size, density, and charge characteristics. Compared with traditional methods, it has the advantages of less sample consumption, shorter separation time and automatic operation, which is especially suitable for high-throughput and rapid isolation of exosomes in clinical applications. However, microfluidic technology still faces some problems in practical applications, such as high cost, low popularity, relatively low device throughput, and no unified standard (31, 32) (**Table 1**).

**Table 1:** Isolation and detection technologies for exosome.

Contents	Platfrom	References
Isolation and purification methods	Ultracentrifugation	(28)
	Sucrose-gradient centrifugation	(33)
	ExoQuick™ test kit	(29)
	Polymer co-precipitation	(34)
	Antibody-coated magnetic bead	(30, 35)
	Ultrafiltration	
	Field flow fractionation	(30, 32)
	Microfluidic chip	(36, 37)
Analysis of morphology and size distribution	Transmission electron microscopy (TEM)	(38)
	Scanning electron microscopy (SEM)	(39)
	Cryo-electron microscopy (cryo-EM)	
	Atomic force microscopy (AFM)	
Analysis of concentration and size distributio	Nanoparticle tracking analysis (NTA)	(40)
	Dynamic light scattering (DLS)	
	Tunable resistive pulse sensing(TRPS)	(41)
Protein quantification and characterization	Western blotting	(38)
	Enzyme-linked immunosorbent assay (ELISA)	
	Mass spectrometry	(42)
Nucleic acid amplification and sequencing	Polymerase chain reaction (PCR)	(43)
	Next-generation sequencing (NGS)	(44)

## 4 The role of exosomes in the liver cancer microenvironment

**Figure 2:**
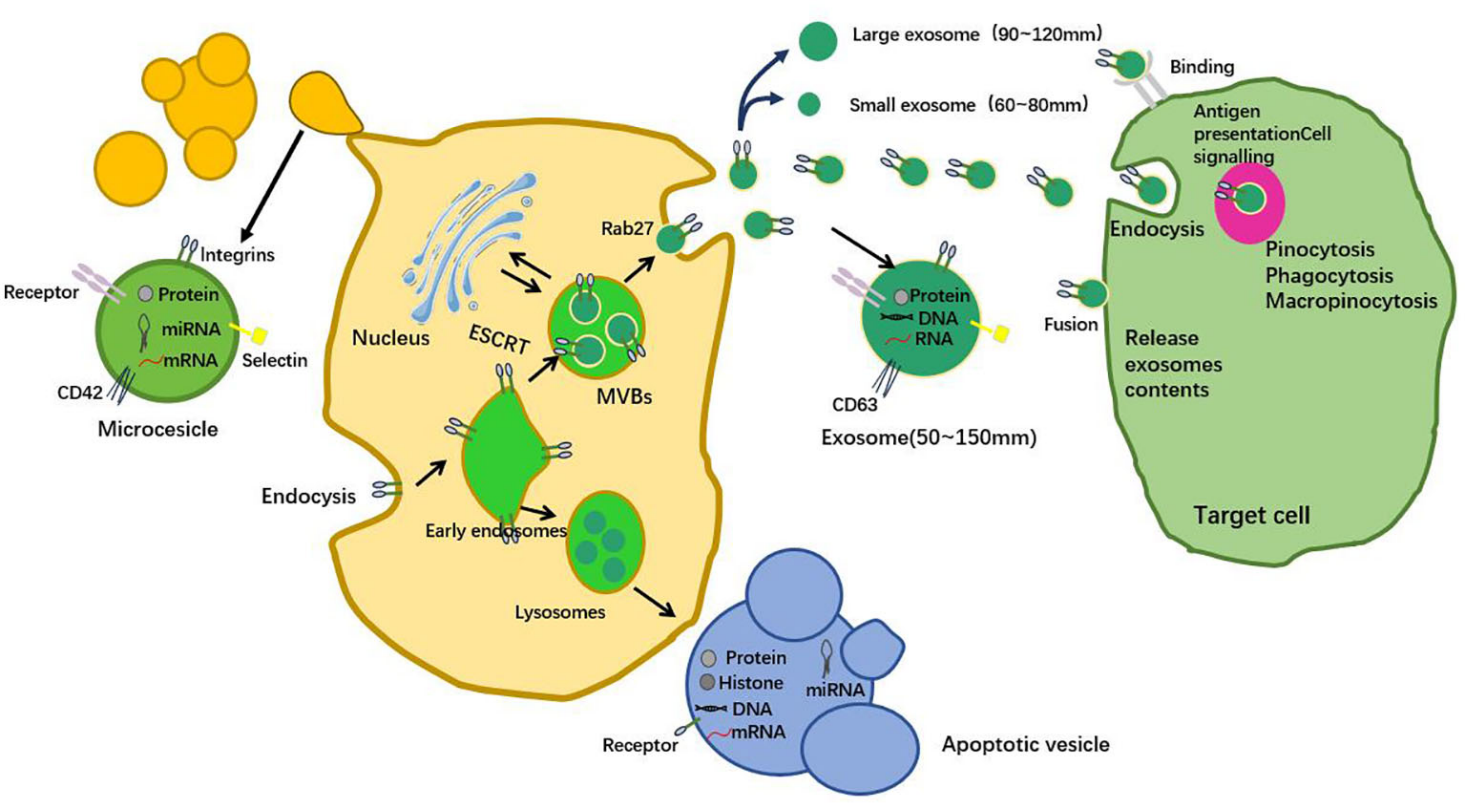
Exosomes, as the medium of intercellular communication, play an important role in the microenvironment of liver cancer. They affect the growth, proliferation, angiogenesis, immune escape, metastasis and invasion of hepatocellular carcinoma cells through the delivery of growth factors, cytokines and other bioactive molecules. Exosomes not only provide a new perspective for the study of liver cancer, but also provide a new potential target for the diagnosis and treatment of liver cancer.

Tumor microenvironment (TME) is a complex environment composed of tumor cells, innate and adaptive immune cells, endothelial cells, cancer-associated fibroblasts, vasculature, and the surrounding matrix, among other cell types (45). The impact of tumors on the microenvironment is complex and varied, including metabolic changes, angiogenesis, immune suppression, and extracellular matrix remodeling. These effects work together to provide favorable conditions for the survival, proliferation, invasion, and metastasis of tumor cells (6). Exosomes can regulate immune cells in the tumor microenvironment through a variety of ways. For example, exosomes secreted by liver cancer cells can bind to PD-1 on the surface of tumor infiltrating lymphocytes (TILs) by carrying PD-L1, and inhibit the activation of T cells, thereby promoting tumor immune escape. In addition, molecules such as microRNA-21 in exosomes can inhibit anti-tumor immune response and promote the maintenance of inflammatory microenvironment by regulating signaling pathways in immune cells, such as NF-κB and STAT3. At the same time, exosomes can also induce the proliferation of immunosuppressive regulatory T cells (Tregs) by carrying cytokines such as transforming growth factor-beta (TGF-β), which further weakens the immune surveillance function (46). Hepatocellular carcinoma has complex and diverse cell-to-cell communication mechanisms in its microenvironment. These communication methods play a crucial role in regulating the occurrence, proliferation, invasion, apoptosis, immune evasion, angiogenesis,EMT of hepatocellular carcinoma cells (47). These cells interact with each other, promoting or inhibiting the occurrence and development of tumors. When tumor cells enter the TME, they stimulate immune cells and other immune-related cells in the TME, promoting immune evasion and tumor progression (48). A recent study has shown that exosomes secreted by hepatocellular carcinoma cells can promote the activation of tumor-associated fibroblasts (CAFs) by carrying specific micrornas, such as miR-1247-3p, and then enhance the invasion and metastasis of tumors (49). In addition, another study found that exosome-mediated communication by carrying lncRNA TUC339 could inhibit natural killer (NK) cell activity in the liver, thereby helping tumor cells to evade immune surveillance and promote tumor growth *in vivo* (50). These *in vivo* studies provided direct evidence for the specific mechanism of exosomes in the progression of HCC, demonstrating the importance of exosomes in regulating the HCC microenvironment and promoting tumor malignant behavior (**Figure 2**).

## 5 Exosomes in liver cancer angiogenesis

Liver tumor-derived exosomes can activate endothelial cells to support liver tumor angiogenesis and thrombosis. At the same time, they can transform fibroblasts and bone marrow mesenchymal stem cells into myofibroblasts, further promoting the generation of liver tumor blood vessels and metastasis (51). Yang et al. studied that HCC-derived exosomal miR-3174 can be transmitted to human umbilical vein endothelial cells (HUVECs) and can induce HCC angiogenesis and increase its ex vivo permeability. It can also promote its metastasis (52).

Targeting exosome-mediated angiogenic pathways may provide an effective complementary strategy for antiangiogenic therapy. Exosomes secreted by liver cancer cells can carry angiogenic molecules, such as VEGF, miR-210, miR-21 and promote angiogenesis by activating signaling pathways in endothelial cells, such as PI3K/AKT and MAPK pathways. These exosome-mediated molecules are different from the targets of existing anti-VEGF therapies and provide new mechanisms of angiogenesis regulation (53–55). Jiang et al. found that miR-30a-5p derived from ICCA can be transferred to endothelial cells and promote their recruitment and proliferation through an exosome dependent mechanism. Further induce angiogenesis and enhance vascular permeability (51).

The conclusions drawn from these various studies suggest that exosome-based strategies have broad application prospects in liver cancer vascular therapy. By adjusting the use of exosomes, a novel, targeted liver cancer treatment may be developed in the future (6, 56).

## 6 Exosomes regulate the growth of liver cancer

Exosomes affect the growth, migration and invasion of tumor cells in the microenvironment of HCC (57).

The PI3K/AKT pathway is one of the most frequently activated growth pathways in liver cancer cells, which can enhance cell proliferation and inhibit apoptosis by regulating cyclin and anti-apoptotic proteins (58). Studies have shown that miR-155 can inhibit SOCS1 through exosomes, and then activate the JAK/STAT signaling pathway to promote the proliferation of liver cancer cells and the inflammatory response in the tumor microenvironment (59). Further studies also revealed the interaction of exosomal mirnas with other tumor-driven pathways such as Wnt/β-catenin and MAPK, whose activation provides growth and invasion advantages to HCC cells (60). Exosomes also play a role in gene expression regulation in HCC. Some research results show that UBXN9 plays a key role in promoting the stimulatory RNA-induced retinoic acid-inducible gene-I-interferon signaling pathway, which can induce effective anti-tumor T cell responses in liver tumorigenesis (61). Higher levels of miR-30a-5p are associated with higher microvascular density (MVD) and poorer prognosis. This suggests that miR-30a-5p may promote the growth and progression of liver cancer by affecting angiogenesis. Further research has found that hypoxia stress can induce the expression of HIF-1α in hepatocellular carcinoma cells, thereby enhancing the expression of miR-30a-5p (51). Additionally, research has found that miR-934 in exosomes derived from tumors can regulate the interaction between CRC cells and TAMs, thereby promoting the occurrence of CRLM (colorectal cancer liver metastasis) (62).

In addition to promoting effect, exosomes also have inhibitory effect on the growth of liver cancer.Hu et al. conducted experiments to study proliferation and migration and found that exosomes released by transgenic tumors had high transfection efficiency and reduced the activity of Wnt signaling pathway, thus inhibiting HCC (63). It has been suggested that some exosomal mirnas may function as tumor suppressors under specific circumstances. Exosomal miR-193-5p can inhibit the growth of HCC by targeting cyclin D1 and inhibiting the MAPK pathway (53). Although exosomes are generally considered promoters of HCC progression, several findings, especially regarding specific mirnas, such as miR-193-5p, suggest potential antitumor effects under specific conditions. Resolving these contradictions will be critical to translating exosome-based therapies into clinical practice.

Based on the relevant studies and experiments, exosomes play a multifaceted role in regulating cancer growth, including promoting tumor proliferation, angiogenesis, immune evasion, and drug resistance. These actions collectively provide favorable conditions for cancer growth and dissemination.

## 7 Exosomes and liver cancer immune escape

The immune system eliminates tumor cells, but some tumor cells are deceptive. Tumor-derived exosomes carry immunosuppressive substances, which are transmitted to immune cells and directly or indirectly inhibit the function of immune cells, thereby accelerating tumor development (64). This discovery determines the crucial role of exosomes in the development of tumor cells. Exosome PD-L1 can bind to the PD-1 receptor on T cells and inhibit the activation and proliferation of T cells, thereby helping tumor cells evade the attack of the immune system (65).

Meanwhile, some studies have found that high levels of exosomal PD-L1 are associated with poor response to immune checkpoint inhibitors(ICIs) such as PD-1/PD-L1 blockade therapy. Patients with elevated exosomal PD-L1 levels showed reduced responsiveness to PD-1 inhibitors, suggesting that these exosomes may serve as biomarkers to predict the efficacy of immunotherapy (53, 66). Many experimental studies have confirmed that by inhibiting the generation of exosomes in cancer and stromal cells, the growth and metastasis process of cancer can be effectively slowed down (67). Furthermore, Lu et al. recently proved that exosomes derived from head and neck squamous cell carcinoma (HNSCC) are taken up by tumor-associated macrophages (TAMs), thereby activating the NF-κB pathway in TAMs, thereby creating an immunosuppressive microenvironment conducive to tumor growth. Therefore, by inhibiting the phagocytic function of macrophages through CD73, it is expected to break the mechanism of tumor immune evasion and improve the therapeutic effect of tumors (68).

Elevated levels of circulating exosomal PD-L1 have been associated with reduced efficacy of ICIs like PD-1/PD-L1 blockers​ (69). Preclinical studies have shown that targeting exosomal PD-L1 can reverse immunosuppression and improve the efficacy of immunotherapy. In mouse models, inhibiting exosome release or neutralizing exosomal PD-L1 restores T-cell function and reduces tumor growth, improving the success of immunotherapy in patients who exhibit resistance (70). Combining exosomal PD-L1 inhibitors with standard immunotherapy is under investigation to improve patient outcomes. The focus of these trials is to assess how targeting exosomal PD-L1 can overcome HCC resistance to PD-1 inhibitors (71).

The ability to measure exosomal PD-L1 by liquid biopsy offers a non-invasive method to monitor the possibility of patient response to immunotherapy. This may allow for more personalized treatment planning, allowing clinicians to predict which patients are likely to benefit most from ICIs or alternative therapies (59). Exosomal PD-L1 not only promotes immune evasion in HCC, but also provides a potential biomarker for evaluating patient response to immunotherapy. This opens new avenues for integrating exosome analysis into clinical decision making for HCC treatment.

## 8 Exosomes and liver cancer metastasis

In the process of tumor cell migration, exosomes act as a key cell-to-cell communication medium, exerting important influence on each link in the chain reaction. This complex process includes the invasion behavior of tumor cells, their survival state in the blood vessels, and attachment and growth with host organs (72, 73).

Recent studies have shown that exosomal miR-3174 plays a key role in HCC progression and metastasis. According to Yang et al., miR-3174 can promote the deterioration of the disease by enhancing vascular permeability and inducing angiogenesis (52). One of the key exosomal miRNAs involved in HCC metastasis is miR-3174. Recent studies have shown that miR-3174 enhances vascular permeability by targeting the tight junction protein ZO-1, disrupting endothelial barrier integrity. This breakdown in vascular structure allows tumor cells to intravasate into the bloodstream, facilitating metastatic spread. Furthermore, high levels of exosomal miR-3174 correlate with increased metastatic potential and poor prognosis in HCC patients (74, 75). Another critical miRNA is miR-21, which is enriched in HCC-derived exosomes. Exosomal miR-21 promotes tumor progression by reprogramming the tumor microenvironment, particularly through its interaction with CAFs. miR-21 drives CAFs to secrete VEGF, enhancing angiogenesis and supporting metastasis (76). Exosomal proteins like TGF-β also contribute to HCC metastasis. Exosomes containing TGF-β can activate stromal fibroblasts, promoting the secretion of matrix metalloproteinases (MMPs) that degrade the extracellular matrix and enhance the invasive ability of HCC cells (77). This exosomal-mediated stromal remodeling is a critical step in enabling metastatic dissemination. Xiaopeng et al. found that under acidic conditions, the metabolic pathway of cancer cells changes, leading to an increase in the number of exosomes released by liver cancer cells and the expression level of functional miRNAs (such as miR-21 and miR-10b), further promoting the proliferation and metastasis of cancer cells (46). Lumin Wang and others found that HOXD3 was proven to target the promoter region of CCR6 and induce its transcription, thereby being delivered to endothelial cells through exosomes and promoting tumor migration. Zhuo-Zhen Lyu and others found that exosomes regulate the expression of miRNAs (such as miR-148a-3p) and target genes (such as MTF-1) by delivering specific non-coding RNAs (such as circ563), thereby affecting the biological behaviors of hepatocellular carcinoma cells (78). Lu et al. found that the level of miR-23a-3p in exosomes derived from M2 macrophages was higher, further promoting the proliferation and metastasis of HCC. These exosomes regulate target genes such as phosphatase and tensin homolog (PTEN) and tight junction protein 1 (TJP1) expression, and stimulate tumor cells to secrete more growth factors and chemokines, thereby promoting the malignant progression of tumors (79). Liu et al. found that exosomes could activate or inhibit specific signaling pathways, such as MAPK/ERK and PDK1/AKT, thereby regulating the proliferation, migration, and metastasis of hepatocellular carcinoma cells (80).

Additionally,EMT has been demonstrated to play a crucial role in the progression of lung cancer from initiation to metastasis. EMT is also associated with various other molecular processes, including tumor immune evasion, as well as the abundance of immune-suppressive cells and the expression of immune checkpoints in liver cancer cells (81, 82). Some studies have indicated that exosomes participate in EMT-related processes by serving as mediators of communication. Huang et al. experimentally identified the pyruvate dehydrogenase complex as a key substrate for activating EMT, demonstrating that the combination of EMT and autophagy inhibitors significantly enhances the therapeutic efficiency of liver cancer *in vitro* and in mice. Exosomes derived from liver cancer cells can induce EMT by activating the TGF-β/Smad signaling pathway, leading to decreased expression of E-cadherin and increased expression of vimentin to promote the migration and invasion of target cells (83). Chen et al. found in their research that exosomes from highly metastatic MHCC 97 H cells can communicate with low-metastatic liver cancer cells, enhancing their role in the EMT process. Furthermore, exosomes derived from liver cancer cells carry β-catenin, thereby inhibiting EMT and metastasis in HCC. Given the pivotal role of exosomes in metastasis, targeting exosome biogenesis and release offers a promising therapeutic approach. For example, GW4869, a neutral sphingomyelinase inhibitor, has been shown to inhibit exosome production. Future studies could explore the efficacy of exosome inhibitors like GW4869 in preventing HCC metastasis *in vivo (* 84).

In summary, exosomes play a crucial role in HCC metastasis by facilitating cell-to-cell communication and promoting key processes such as angiogenesis, tumor cell migration, and EMT. Specific exosomal miRNAs, including miR-3174 and miR-21, have been identified as major drivers of metastasis (85). miR-3174 enhances vascular permeability to promote tumor cell dissemination, while miR-21 reprograms the tumor microenvironment by interacting with CAFs to stimulate angiogenesis (86). Additionally, exosomal proteins like TGF-β contribute to stromal remodeling and increased tumor invasiveness. Exosomes also regulate key signaling pathways such as MAPK/ERK and PDK1/AKT, further driving the metastatic progression of HCC (87). Targeting exosome biogenesis and release, as well as specific exosomal cargo like miR-3174 and miR-21, presents a promising therapeutic approach to prevent HCC metastasis (88).

## 9 Exosomes as diagnostic and prognostic biomarkers in liver cancer

Nowadays, exosomes are being used as valuable biomarkers for diagnosing and predicting various types of cancer due to their own properties and composition. The exosomes contain a variety of functional molecules, including proteins, lipids, DNA, various RNAs, and metabolites (89). Among these, proteins and ncRNAs are the most abundant substances in exosomes. Therefore, when referring to them as biomarkers, their value and utility are greater than those of other substances and are more widely used (90).

Under the protection of phospholipid bilayer membrane, exosomes cannot be degraded by any enzyme, and exosomes are considered to be suitable diagnostic tools.Researcher Lee et al. reported that the lncRNA-ATB contained in the serum exosomes of HCC patients was positively correlated with the TNM staging and volume of the tumor and negatively correlated with OS (91). Sohn et al. found that the levels of miR-18a, miR-221, miR-222, and miR-224 in the exosomes produced by HCC patients were lower than those of HBV patients.Therefore, exosomes can be used as independent markers for the diagnosis and prognosis of HCC.

In the detection of exosomes, several studies have used nucleic acid detection methods that can distinguish between patients and healthy individuals by detecting samples from plasma. Although this detection method is more expensive, it still has great potential in clinical applications (92). The clinical and research experiments mentioned above all indicate that exosomes can serve as reliable biomarkers for cancer detection and diagnosis of its type (84). Exosome proteins have a greater potential for use in cancer diagnosis, while exosome non-coding RNA plays a more important role in predicting various types of cancer. Their role in carrying specific molecular signatures, such as miRNAs, proteins, and non-coding RNAs, makes them valuable tools for monitoring disease progression and response to treatment. However, while their potential is compelling, several challenges must be addressed to translate these findings into routine clinical practice.

One of the key challenges in utilizing exosomes as clinical biomarkers is the standardization of detection methods. Currently, exosome isolation techniques such as ultracentrifugation, size-exclusion chromatography, and immunoaffinity capture vary widely in terms of efficiency, purity, and reproducibility (93). Another significant challenge is the **v**ariability in exosome content among patients. Exosomal cargo is influenced by several factors, including tumor heterogeneity, disease stage, and even the patient’s immune response, which may result in significant variability in exosomal biomarkers across individuals (94). Furthermore, there is the issue of EV heterogeneity, where exosomes are just one subtype among many EVs. This complicates biomarker studies, as differentiating between exosomes and other EVs is challenging, yet crucial for the specificity of diagnostic tests (95) (**Table 2**).

**Table 2:** Exosomes as diagnostic and prognostic biomarkers in liver cancer.

Exosomal Cargos	Method	Clinical significance	Reference
Exosomal proteins (VCAN, TNC, THBS2, PD-L1)	By comparing the proteomic profiles of tumor tissue and plasma-derived EVs, the cancer-specific protein signatures of EVs can be identified	EV proteins can be used as reliable biomarkers for cancer detection and diagnosis of cancer types	(90)
miRNA (miR-21, miRNA-373, miR-1290, miR-19 a, miR-221, miR-122- 5 p)	The levels of different miRNAs within exosomes are associated with different cancer cells	Different miRNAs of exosomes have been detected as biomarkers in the plasma of cancer patients.	(96–98)
lncRNA (lncRNA-ATB, lncRNA-UCA 1, HOTTIP)	lncRNAs are selectively sorted into cancer exosomes	lncRNA can be used as a potential diagnostic biomarker for liver cancer	(91, 99)
circRNA	circRNA can be identified at levels in exosomes	circRNA can be used as a potential diagnostic biomarker for liver cancer	(100)
dsDNA	Double-stranded DNA (dsDNA) is characterized in tumor-derived exosomes	dsDNA can be used as a potential diagnostic biomarker for liver cancer	

Although AFP is widely used in clinical practice, it has significant limitations in terms of sensitivity and specificity, especially in early-stage HCC. Exosome-based biomarkers have been shown to be superior to conventional serum AFP in detecting early HCC and predicting outcome. For example, exosomal mirnas such as miR-21, miR-122, and miR-155 can provide insight into tumor behavior and perform noninvasive detection with higher diagnostic accuracy (101–103). However, there are many challenges to integrating exosome biomarkers into routine clinical practice. In contrast, AFP testing is mature, cost-effective, and widely available, but it often fails to detect early-stage HCC and can produce false positives, especially in patients with chronic liver disease (104–106) (**Table 3**).

**Table 3:** Exosomes-based biomarkers of liver cancer were directly compared with traditional biomarkers such as AFP.

Biomarkers	Advantages	Limitations	Reference
Exosome-based biomarkers	High sensitivity and specificity for early HCC detection	Lack of standardization of isolation and detection methods	(92, 102, 103)
	Real-time tumor dynamics were reflected	Differences in exosome content between patients	
	It is possible to monitor treatment response		
AFP	Widely available	The sensitivity is low, especially for early HCC	(104–106)
	Mature and cost-effective	False positive rates are high, especially in patients with liver disease	

## 10 Exosomal microRNA

Some researches have found that microRNAs are a key mechanism component of tumor-derived Exosomes in executing their functions, and exosomes have been determined as an important medium for communication between tumor cells and the microenvironment (107). Exosome microRNAs are highly abundant in exosomes and are associated with immunoregulation, chemotherapy resistance, and metastasis of various tumor types (108). The influence of exosome microRNA on the liver microenvironment and its role in GC-LM are significant. Some researchers have found that miRNAs account for about 43% of the RNA in exosomes, and play an important role in the biological regulation function of exosomes. Exosome miRNA has also been proven to participate in organ-specific metastasis of various cancers, including lung cancer, breast cancer, pancreatic cancer, and melanoma, by reshaping the target organ microenvironment (109).

Exosome microRNA has been applied in the clinical treatment of autoimmune diseases. A related study shows that the exosomal microRNA released by Tlymphocytes in rodents and humans can transfer to beta cells in an active form, leading to beta cell apoptosis (110). According to recent research findings, exosomes can promote cancer progression by mediating miRNA communication between tumor cells and surrounding cells, thereby affecting tumor angiogenesis. In addition, detecting circulating exosomal miRNA in serum for cancer diagnosis has been proven to be a reliable method.

Exosomal miRNAs are heavily implicated in HCC’s progression by regulating gene expression in recipient cells. For example, miR-21 in HCC-derived exosomes is known to facilitate tumor progression by transforming hepatic stellate cells into CAFs, which then promote tumor growth and metastasis (86). Another significant miRNA, miR-122, a liver-specific miRNA, is under-expressed in HCC exosomes. Restoring its expression has been shown to suppress tumor growth by regulating metabolic pathways, such as the Warburg effect, and inhibiting the PI3K/Akt pathway (111).

**Figure 3:**
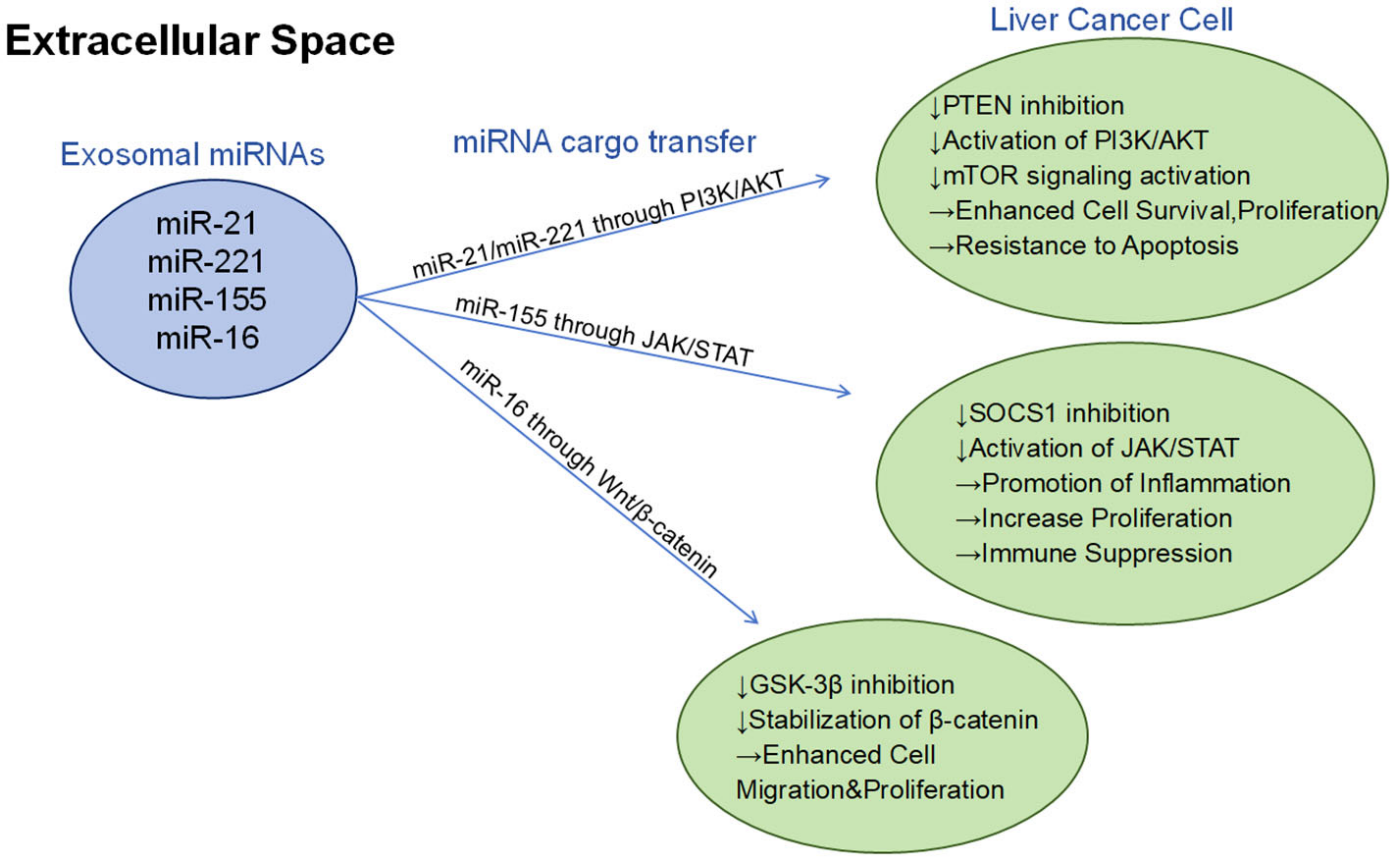
Different exosomal mirnas (such as miR-21, miR-221, miR-155, and miR-16) regulate the growth, survival, migration, and immune escape mechanism of hepatocellular carcinoma cells through three major signaling pathways (PI3K/AKT, JAK/STAT, and Wnt/β-catenin):PI3K/AKT pathway: By inhibiting PTEN, activation of the PI3K/AKT pathway further activates mTOR signaling, thereby enhancing cell survival, proliferation, and leading to anti-apoptotic properties. JAK/STAT pathway: By inhibiting SOCS1, the JAK/STAT signaling pathway is activated to promote inflammatory response and cell proliferation, while inhibiting immune function. Wnt/β-catenin pathway: by inhibiting GSK-3β and stabilizing β-catenin, it enhances cell migration and proliferation.

Combining the above review, microRNA provides diagnostic biomarkers and valuable therapeutic targets, which can serve as potential diagnostic or prognostic biomarkers in the tumor microenvironment and have great potential (112) (**Figure 3**).

## 11 Exosomal proteins

Exosome proteins are proteins that are packed inside or embedded on the surface of exosome membranes, and are an extremely important exosome transport protein (7). Exosome proteins have become a potential important source of biomarkers for observing exosome production, targeted therapy, and cancer diagnosis and prediction. Exosomal proteins also play a role in regulating the treatment of tumor-related processes (113). Many experiments have demonstrated that exosomal proteins play an important role in tumor therapy, angiogenesis, epithelial-mesenchymal transformation, tumor microenvironment and drug resistance research. However, the interaction mechanism between exosomal proteins and hepatocellular carcinoma therapy is rarely studied (114).

Exosomal proteins consist of various types, including MHC, tetraspanins, various enzymes, glycoproteins, as well as a range of ligands and receptors (21). These exosomal proteins provide sufficient diagnostic, monitoring and prognostic information for the treatment of liver cancer (115). Arbelaiz et al. compared the protein levels in exosomes from liver cancer patients and healthy cohorts in the study and found that exosomal G3 BP can better diagnose liver cancer and differentiate it from other liver diseases. Fu et al. reported in their study that SMAD 3 protein is present in exosomes derived from liver cancer cells and is positively correlated with the pathological grade of liver cancer. Wang et al. reported in their study that the level of 14-3-3 protein in exosomes derived from liver cancer cells increases, and 14-3-3 protein can weaken the anti-tumor activity of T cells.

Exosomal proteins play a critical role in intercellular communication and remodeling the tumor microenvironment. For instance, proteins like HSP70 and EGFR are highly expressed in HCC-derived exosomes, promoting cancer cell migration and invasion (116, 117). These proteins also contribute to the development of drug resistance by altering the surrounding stroma, making them key players in HCC progression and therapeutic resistance.

The most important role of exosome proteins in cancer treatment is drug delivery. By engineering the surface proteins of exosomes, drugs and therapeutic agents can be directly packaged into exosomes, thereby achieving higher efficiency (118). Xu et al. evaluated in their paper that the immune function of exosomes may make them specific drug delivery tools or vaccines for cancer immunotherapy (119). In summary, exosome proteins play a key role as biomarkers in the prediction and diagnosis of various cancers, can be used as drug delivery carriers to selectively deliver drugs to designated cell receptors, and have good potential applications in these areas. However, the knowledge of the functions and applications of exosome proteins is still limited.

## 12 Exosomal lncRNA

There are already some long non-coding RNAs (lncRNAs) that have been evaluated as playing important roles in the carcinogenesis and progression of liver cancer. The exosomes carrying lncRNAs from HCC cells have been identified as key mediators for the treatment of HCC (120). lncRNA H19, transferred via exosomes, enhances the invasiveness of HCC cells by interacting with miRNAs and influencing the tumor microenvironment (121). Similarly, lncRNA TUC339 promotes the proliferation and migration of HCC cells by modulating various signaling pathways (122).

Wang et al. found in their study that a novel lncRNA was overexpressed in hepatocellular carcinoma by using qRT-PCR and fluorescence *in situ* hybridization, and it was positively correlated with the predictive index of cancer, thus being a novel oncogenic lncRNA (123). Furthermore, M. Xu et al. confirmed in their experiment that incMMPA (an lncRNA) could regulate the tumor of HCC through immunohistochemical analysis and fluorescence, and it could increase the proliferation of HCC cells by interacting with miR-548 s *in vivo* (124). H. Cha et al., in their study, also evaluated a novel regulatory factor that modulates hepatocarcinogenesis through lncRNA-HEIH, which may serve as a potential therapeutic target for hepatocellular carcinoma (125). In summary, lncRNAs from exosomes have great potential for the proliferation of hepatocellular carcinoma and its treatment, and can be used as a key mediator for the treatment of liver cancer.

## 13 Exosomal lipids

The exosomal lipids refer to the biologically active lipids carried by exosomes, which have great potential to be used as liver delivery vehicles and cancer agents targets. Exosomal lipids contribute to both the structural stability of exosomes and the regulation of metabolic processes in recipient cells. Lipids like **s**phingomyelin and cholesterol are known to facilitate communication between HCC cells and are implicated in drug resistance mechanisms. By influencing metabolic pathways, exosomal lipids could offer new biomarkers for early detection and therapeutic targets (126). Both artificially synthesized liposomes and naturally derived exosomal lipids are ideal drug delivery carriers (127). In addition, exosomal lipids have good functionalities in targeted therapy and natural affinity to liver cells (128). Some studies have evaluated the above functions of exosomal lipids.

Tabernero and colleagues first achieved liver cancer targeted therapy by using exosomal lipids as drug delivery carriers in phase I studies. Some researchers evaluated in the experiment the ability of exosomal lipids loaded with miR-375 and sorafenib to inhibit autophagy process and reduce tumor burden to achieve high efficiency and stability in treatment (129). Woitok et al. delivered exosomal lipids loaded with siRNA targeting Jnk 2 to mice with chronic liver disease, showing a reduction in precancerous nodules in liver cells and a benign shift in the cancer microenvironment (130).

In conclusion, exosomal cargo, including micrornas, proteins, lncrnas, and lipids, plays a multifaceted role in HCC biology and progression. Exosomal micrornas contribute to immune regulation, chemotherapy resistance and metastasis in regulating gene expression. For example, miR-21 and miR-122 affect HCC growth and tumor microenvironment (86, 111). Exosomal proteins such as HSP70 and EGFR are involved in cancer cell migration, invasion and drug resistance, and can be used as potential biomarkers for diagnosis and targeted drug delivery (116, 117). Exosomal lncrnas, such as H19 and TUC339, enhance HCC cell invasiveness and proliferation by interacting with mirnas and altering signaling pathways (122). Finally, exosome lipids contribute to the structural stability of exosomes and regulate the metabolic processes of recipient cells, which are promising targets for drug delivery and liver-specific therapy. Collectively, these exosome components provide a dynamic network that influences HCC progression, providing potential diagnostic biomarkers and therapeutic targets (126).

## 14 The role of exosomes in liver cancer therapy

In the treatment of liver cancer, exosomes are used to deliver RNA complexes of viruses to liver cells, which are then used by T cells to suppress immune responses. And exosomes can be used as independent tumor markers in the assessment of HCC staging, efficacy, and prognosis. Therefore, exosomes play a key role in the occurrence, diagnosis, and treatment of HCC and have broad clinical applications (131).

Some researchers have shown that exosomes can affect the growth and metastasis of liver cancer cells by secreting substances for regulation (3). Additionally, some researchers have found that, in addition to liver cancer cells, other cells can also secrete exosomes to promote the growth of liver cancer cells and reduce DNA damage (132).

Camel milk exosomes show therapeutic potential, particularly in slowing breast cancer progression by inducing apoptosis and reducing oxidative stress, inflammation, and metastasis, with local injection proving more effective than oral use.They also enhance the effects of tamoxifen in chemotherapy and, when combined with chitosan nanoparticles and sorafenib, significantly reduce tumor burden in cancer models. Additionally, camel milk proteins exhibit immunomodulatory and antioxidant properties (15, 133–138). Curcumin and ginger-derived exosomes reduce inflammatory cytokines in the tumor microenvironment, suppressing cancer progression (57). In the treatment of liver cancer, Tcm-derived exosomes can inhibit cell proliferation by regulating cell cycle and inducing apoptosis, anti-inflammation by inhibiting pro-inflammatory cytokines and regulating immune response, anti-oxidation by scavenging free radicals and regulating antioxidant enzyme activity, and reducing liver fibrosis by inhibiting hepatic stellate cell activation and regulating extracellular matrix metabolism (139–142).

Therefore, using exosomes to trigger anti-tumor immunity has become a promising therapeutic strategy (142). Exosomes play a key role in the occurrence, diagnosis, and treatment of hepatocellular carcinoma, and many of its substances, such as mRNA, can serve as new biomarkers, laying a foundation for subsequent research and clinical applications.

### 14.1 Exosome-based immunotherapy

**Figure 4:**
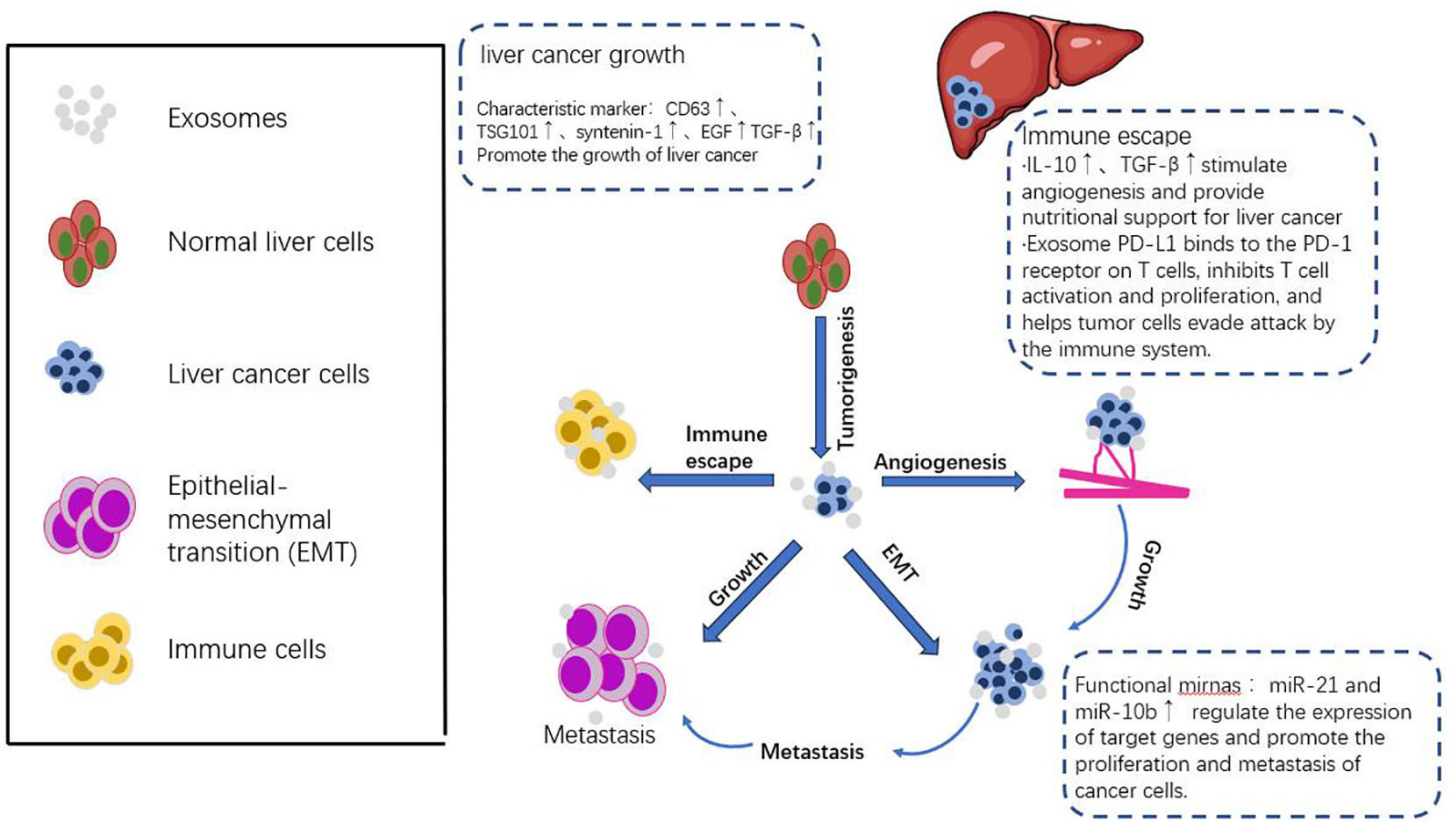
The dual mechanism of exosomes in tumor immune response. The mechanism of anti-tumor immune response involves the binding of specific new antigens such as HSP70 and MART1 to the extracellular matrix of the tumor by the extracellular matrix outside the tumor, which is then presented to dendritic cells (DC) or directly stimulated by T cells. These extracellular matrices not only promote the expression enhancement of CD80, CD86 and MHC-II on the surface of dendritic cells, thereby effectively activating CD4+ T cells, but also activate DC and CD8+ T cells directly through the exosomal DNA they carry. Furthermore, tumor extracellular matrices induce the activation of natural killer cells and macrophages by releasing heat shock protein 70, thereby enhancing the anti-tumor immune response. In particular, dendritic cells release exosomes containing antigens and MHC-I complexes, which are crucial for activating cytotoxic T cells and help suppress the growth and spread of tumors. However, tumor extracellular matrices also exhibit pro-tumor effects in the immune system. They weaken the immune system’s ability to strike at the tumor by suppressing the function of dendritic cells, T cells, and natural killer cells, while increasing the number of myeloid-derived suppressor cells (MDSCs) and Tregs. In addition, tumor extracellular matrices carry PD-L1 molecules from tumor cells and transfer them to dendritic cells or macrophages, further blocking T cell activation and function, providing favorable conditions for the growth and spread of tumors. This dual action mechanism reflects the complexity of the tumor exosome in the tumor immune response and provides a new perspective and strategy for tumor immunotherapy.

Some studies have concluded that exosomes play a regulatory role in the immune response. The substance that exerts an inhibitory effect is TDE, which is mainly found in exosomes. TDE has been shown to have immunosuppressive effects and promote tumor growth (143). Zhi et al. explained in their research paper that exosomes derived from tumor cells, dendritic cells, B lymphocytes, T lymphocytes, and natural killer cells have immune stimulating and inhibitory effects in the immune response (119). Furthermore, exosome-based immunotherapy has been applied to animal models in previous studies. Because exosomes have the ability to carry various immune-suppressive signals, they have great potential as cancer biomarkers (144). Another study shows that the accumulation of PD-L1 on exosomes can be used as a predictive factor for the effectiveness of NSCLC immunotherapy, conveying diagnostic and prognostic information (145). The above studies have shown that exosomes can be used as biomarkers through many different components and have great potential for immune response, but there are still some problems that need to be solved (**Figure 4**).

### 14.2 Exosomes serve as anticancer drug delivery carriers

In clinical treatment, the therapeutic effect of anti-cancer drugs is limited by the properties of the drugs themselves, such as poor solubility and short half-life, the limitations of drug transport and action pathways, leading to cancer cell resistance and significant local and systemic toxicity, resulting in unsatisfactory treatment effects (146).

Researchers have tested various formulations to address these clinical challenges, and although these formulations have shown different promise in clinical trials, they all have limitations (147). More and more researchers and research results show that exosomes are a promising drug delivery vehicle with natural advantages. So researchers have turned their attention to exosomes (148).

Many studies have evaluated the advantages of exosomes as drug delivery vehicles. First, Schindler et al. concluded in their study that exosomes are able to enter cells and internalize and distribute drugs faster than general drug carriers. They also found that exosomes have the highest intracellular accumulation and cytotoxicity in tumor cells compared to other formulations. In the process of drug transportation, exosomes selectively deliver drugs to cancer cells, with the least toxicity to normal cells (149). The various properties of exosomes support their application in anti-cancer therapy. Kim et al. used exosomes as a drug carrier for immunosuppressive treatment in their study and evaluated the treatment effect. The results showed that tumor growth was completely inhibited without significant side effects (150). Additionally, exosomes can be customized as precise carriers targeting tumor cells, providing new possibilities for cancer treatment (151). However, exosomes are prone to degradation and aggregation during isolation, storage, and drug delivery, which can impair their integrity and therapeutic efficacy. Therefore, it is essential to develop the best storage conditions and preservation methods, such as freeze-drying and cryopreservation. However, these techniques may alter the exosome membrane or affect the cargo release curve, so further studies are needed to maintain stability without compromising function (152). Exosomes are prone to degradation and aggregation during isolation, storage, and drug administration, which can compromise their integrity and therapeutic efficacy However, there are challenges in ensuring the safety and targeting specificity of exosomes. For example, while Mir-210-loaded exosomes show promise in reducing ischemia-reperfusion injury, there is still concern about off-target effects when exosomes are administered systemically (53). Exosomes can be taken up by non-target cells due to their inherent ability to fuse with various cell types, leading to unintended delivery of therapeutics and possible side effects (153, 154).

By modifying the surface of exosomes, researchers aim to enhance their ability to deliver therapeutic agents directly to tumor cells, minimizing off-target effects. One strategy involves the use of ligand-receptor interactions; for example, exosomes can be engineered to express ligands or antibodies on their surface that specifically bind to receptors overexpressed on HCC cells (155). Another approach includes the insertion of peptides or aptamers on the exosomal membrane to increase binding affinity to target cells (156).

Combining exosome-based drug delivery with existing therapeutic modalities, such as chemotherapy, radiotherapy, and immunotherapy, has shown promising results. For instance, exosomes loaded with chemotherapeutic agents, like doxorubicin, can be combined with immune checkpoint inhibitors to enhance the anti-tumor immune response and improve therapeutic outcomes (157). An innovative strategy involves loading exosomes with specific therapeutic agents such as siRNA, miRNA, and CRISPR-Cas9 components to target oncogenic pathways in HCC cells. By using electroporation or chemical transfection methods, these genetic materials can be efficiently loaded into exosomes and delivered to target cells (158).

Although many researchers have conducted extensive and detailed studies to prove that exosomes have great potential as drug carriers in liver cancer treatment, there are still some problems in terms of technology, function, and safety that need to be solved. Therefore, more research is needed to find solutions to these problems.

## 15 Conclusion

In summary, the application of exosomes in liver cancer has attracted much attention in the field of tumor therapy. Exosomes can deliver drugs to target organs and cells in the body more efficiently by encasing drugs in exosomes, thereby reducing side effects and enhancing therapeutic effectiveness. In this paper, we review that exosomes play a multifaceted role in regulating cancer growth, including promoting tumor proliferation, angiogenesis, immune evasion, and drug resistance, actions that together provide favorable conditions for cancer growth and spread. Therefore, the study of exosomes is of great significance for revealing the pathogenesis of cancer and developing new therapeutic strategies.

In addition, cell-derived exosomes can be used to estimate biomarkers for various cancer diagnoses, predictions, therapeutic efficacy, and outcomes. Exosomal RNA, DNA, proteins, lipids and other substances provide valuable therapeutic targets for the treatment of hepatocellular carcinoma and serve as potential diagnostic or prognostic biomarkers in the microenvironment of liver cancer. However, the current understanding of the function and application of exosomal proteins is still limited. Exosomes still have some technical, functional and safety issues that need to be addressed. Firstly, the development of more reliable and efficient exosome isolation techniques is the key to ensure high purity and yield, which is essential for basic research and clinical translation. Secondly, exploring the heterogeneity of exosomes is crucial to fully understand their different roles in liver cancer and to exploit this diversity to obtain treatment. Finally, the clinical translation of exosome-based therapies needs to be rigorously validated in human trials to address issues of safety, efficacy, and scalability.

Despite these challenges, the prospect of using exosomes for the treatment of liver cell tumors is still very promising. As technology continues to advance, these challenges will be overcome one by one, and exosomes will become an important tool for tumor treatment. It is believed that exosomes will be widely used in the treatment of liver cancer in the future.
